# The Ellipsoid Zone Is a Structural Biomarker for Visual Outcomes in Diabetic Macular Edema and Macular Hole Management

**DOI:** 10.3390/vision9010004

**Published:** 2025-01-13

**Authors:** Shivani Chaturvedi, Amisha Paul, Samya Singh, Levent Akduman, Sandeep Saxena

**Affiliations:** 1 Department of Ophthalmology, King George’s Medical University, Lucknow 226003, India; shivani.c4u@gmail.com (S.C.); samya11sept@gmail.com (S.S.); 2 EyeCare Partners, St. Louis, MO 63117, USA; amisha.paul@health.slu.edu

**Keywords:** ellipsoid zone, structural biomarker, spectral-domain optical coherence tomography, vascular endothelial growth factor, diabetic macular edema, macular hole surgery

## Abstract

Objectives: The goal was to study the ellipsoid zone (EZ) as a structural biomarker for final visual outcomes after pharmacological intervention in center-involving diabetic macular edema (DME) and surgical intervention for full-thickness macular holes (FTMHs). Methods: This was a tertiary care center-based retrospective study. After sample size calculations, data from 64 consecutive cases were collected, with subjects aged between 40 and 60 years. Thirty-two cases of DME with anti-vascular endothelial growth factor (VEGF) therapy and 32 cases of FTMHs with successful macular hole surgery (MHS) were studied. Spectral-domain optical coherence tomography (SD-OCT) data were collected. Measurements of EZ defects documented at the time of presentation and 12 weeks after intervention were analyzed using the caliper function of the machine. EZ restoration was graded, and a Pearson correlation analysis was performed. Results: Mean logMAR VA decreased after intravitreal therapy (IVT) from 1.12 ± 0.22 pre-intervention to 0.81 ± 0.41 post-intervention and after MHS, from 1.05 + 0.25 to 0.62 + 0.11 (*p* < 0.001). EZ disruption reduced from 73.4% to 19.4% after IVT and from 67% to 19.3% after MHS. DME and MHS postoperative visual acuity and residual EZ defect were observed to have a statistically significant linear correlation (r = 0.794, *p* < 0.001 and r = 0.894, *p* < 0.001, respectively). The EZ was found to be an excellent structural biomarker for final BCVA (area under curve = 0.95 for DME and 1.00 for MHS). Conclusion: Notable EZ restoration results were obtained from pharmacological and surgical interventions. The EZ proves to be a critical structural biomarker for predicting visual outcomes in center-involving DME and MHS.

## 1. Introduction

Spectral-domain optical coherence tomography (SD-OCT) is the most widely used and accurate diagnostic instrument for defining the in vivo histology of the retina [1]. This technological advancement has significantly advanced our comprehension of DME and FTMHs. Retina photoreceptor integrity defines the visual outcome of retinal disease. The ellipsoid zone (EZ) is demarcated as a hyper-reflective band below the external limiting membrane (ELM), which clinically represents photoreceptor integrity. Diabetic macular edema (DME) and full-thickness macular holes (FTMHs) are among the most significant medical and surgical diseases of the retina where EZ disruption occurs.

The global prevalence of DME is estimated to be 6.8%, affecting approximately 27 million adults worldwide [2]. The main factor causing DR is extended exposure to elevated blood sugar levels in individuals with diabetes mellitus (DM), leading to the formation of advanced glycation end-products (AGEs). These AGEs trigger the expression of vascular endothelial growth factor (VEGF) [3]. Multiple physiological and pathological effects can be attributed to VEGF [4,5]. An increase in VEGF has been related to the increased disruption of the EZ and increased central subfield thickness (CST) in DME [6]. Our previous studies have shown that the administration of intravitreal anti-VEGF agents is associated with the restoration of the EZ and subsequent improvement in VA [7,8].

An FTMH is a pathological defect of the macula, spanning through all layers of the retina—from the internal limiting membrane (ILM) to the retinal pigment epithelium (RPE). Individuals with FTMHs commonly suffer from notable visual impairment, metamorphopsia, and central scotomas [9,10,11]. An FTMH is typically addressed through surgical intervention, employing a three-port pars plana vitrectomy combined with gas or air tamponade, often accompanied by ILM peeling. The current success rate for closing an FTMH exceeds 90% [12,13]. Factors such as the duration of symptoms, preoperative macular hole size, preoperative visual acuity, axial length, age, and sex have been identified as potential prognostic indicators [14]. The advent of SD-OCT has revolutionized our ability to analyze and visualize each layer of the retina and its structures in vivo.

The WHO has defined a biomarker as any substance, structure, or process that can be measured in the body and can predict the incidence or outcome of the disease [15]. This study was undertaken to review the role of the EZ as a structural biomarker after pharmacological and surgical intervention in DME and FTMH, respectively.

## 2. Materials and Methods

This study’s authors confirm their adherence to the doctrines of the Declaration of Helsinki. This study was a retrospective study, and all patients’ clinical, biochemical, and retinal diagnostic data were retrieved from computerized records. In accordance with the ECR/262/Inst/UP/2013/RR-19, ethical approval was waived due to the retrospective nature of the study.

### 2.1. Sample Size Calculation

The sample size was calculated using the formula *n* = (t_n-1_, α/2 + t_n-1_, β)^2^/d^2^ where d = delta/standard deviation, α = probability of detecting a false effect, β = 1-power (probability of detecting a true effect) and t = student t quantile with v degrees of freedom and probability p. n is rounded to the closest integer. Based on this, a sample size of 32 was estimated, with power of 90%.

### 2.2. Patients

This tertiary care center-based retrospective study included data analysis of a total of 64 subjects aged between 40 and 60 years. A total of 32 cases of type II DM with center-involving DME and EZ disruption were studied after intravitreal therapy (IVT) [intravitreal bevacizumab (1.25 mg/0.5 mL); 3 consecutive injections at a one-month interval]. Thirty-two consecutive cases of successful MHS for FTMHs were studied in patients who had undergone pars plana vitrectomy with brilliant blue-assisted ILM peeling with gas instillation and were in a face-down position for seven days. Patients with ocular conditions—uveitis, age-related macular degeneration (including drusen, pigment epithelial detachment, and EZ atrophy), or other retinal diseases associated with macular edema, such as retinal venous and arterial occlusive disease—were excluded. Additionally, those with systemic disorders that could influence retinal vasculature—chronic kidney disease, pulmonary conditions like pulmonary embolism, pulmonary hypertension, or essential hypertension—were also excluded from the study. Patients with a history of previous intravitreal injections, laser treatments, and surgical interventions like vitreoretinal or glaucoma surgery were not included.

### 2.3. Data Collection

The patient’s age and sex were documented. The SD-OCT of 64 patients with pre-intervention and 12-week post-intervention follow-up data were analyzed. Manual Caliper, software tool (version- 11.5.2.54532) of SD-OCT (The Cirrus HD-OCT 6000 (Carl Zeiss Meditech, Inc., Dublin, CA, USA)) was used. A macular cube (128 × 512) with a horizontal 5-line raster scan (signal strength > 7), fovea-centered, was acquired to measure EZ defects. EZ disruption was present in the subfoveal region, with parafoveal extension in some cases. The study’s endpoint was the evaluation of the status of EZ 12 weeks after intervention.

SD-OCT assessment of EZ was conducted by two independent observers masked to the pre- or post-intervention status. The inter-observer correlation was computed by the Spearman correlation coefficient.

Image Interpretation: The EZ was demarcated as the first hyper-reflective band above the RPE. The ELM was defined as a discrete hyper-reflective band at the outermost border of the outer nuclear layer, located above the EZ.

Analysis was performed based on a unique EZ restoration grading system after measurements of EZ defects pre- and post-intervention: Grade 0: minimal EZ restoration; Grade 1: partial EZ restoration; and Grade 2: complete EZ restoration.

### 2.4. Data Analysis

Continuous data were depicted as means ± SE (standard error of the mean). Discrete (categorical) data were depicted in numbers (*n*) and percentages (%). Two continuous independent groups were compared by paired *t*-test. Continuous groups were compared by analysis of variance (ANOVA), and the significance of the mean difference within (intra) and between (inter) the groups was determined by the Tukey HSD post hoc test. ANOVA was used to prove that the three grades of classification are statistically significant and corroborate the visual outcome. A two-tailed result (α = 2) *p* < 0.05 was considered statistically significant. The receiver operating characteristic (ROC) curve area under curve (AUC) was used to evaluate the EZ as the biomarker for the final BCVA. Analyses were performed on SPSS software (Windows version 24.0).

## 3. Results

The demographic characteristics and qualitative variables before and after intervention in DME (Table 1) and FTMH (Table 2) patients have been summarized below. In the DME group, the disorganization of retinal inner layers (DRIL) was observed in 12.5% of cases. However, large intraretinal cysts were not observed.

Age did not display a statistically significant difference across the study groups (*p* > 0.05). The one-way ANOVA revealed a notable and statistically significant enhancement in the final BCVA for both study groups (*p* < 0.001).

**Figure 1:**
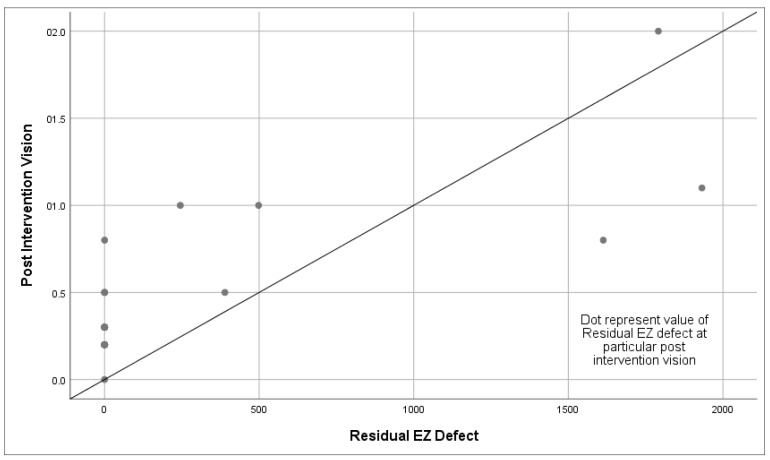
Statistically significant linear relationship between post-interventional visual acuity and residual ellipsoid zone defect in diabetic macular edema (*n* = 32).

**Figure 2:**
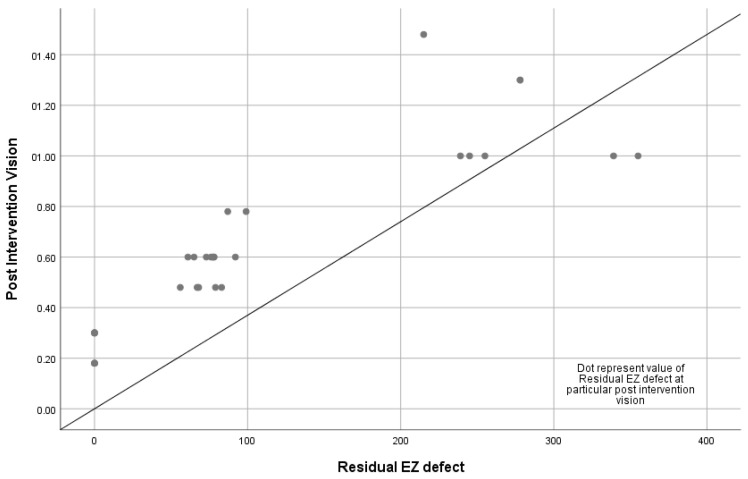
Statistically significant linear relationship between post-interventional visual acuity and residual ellipsoid zone defect in full-thickness macular holes (*n* = 32).

Pearson correlation analysis was performed to assess the connection between the residual EZ defect observed on SD-OCT and the final BCVA in patients with DME (r = 0.794, *p* < 0.001) (Figure 1) and FTMHs (r = 0.894, *p* < 0.001) (Figure 2) after intervention. The analysis revealed a significant linear relationship between the residual EZ defect and the post-interventional final BCVA.

The binary classifier model categorized outcomes into two states—good post-interventional visual acuity and poor post-interventional visual acuity—based on the residual ellipsoid zone defect in both DME and FTMHs.

**Figure 3:**
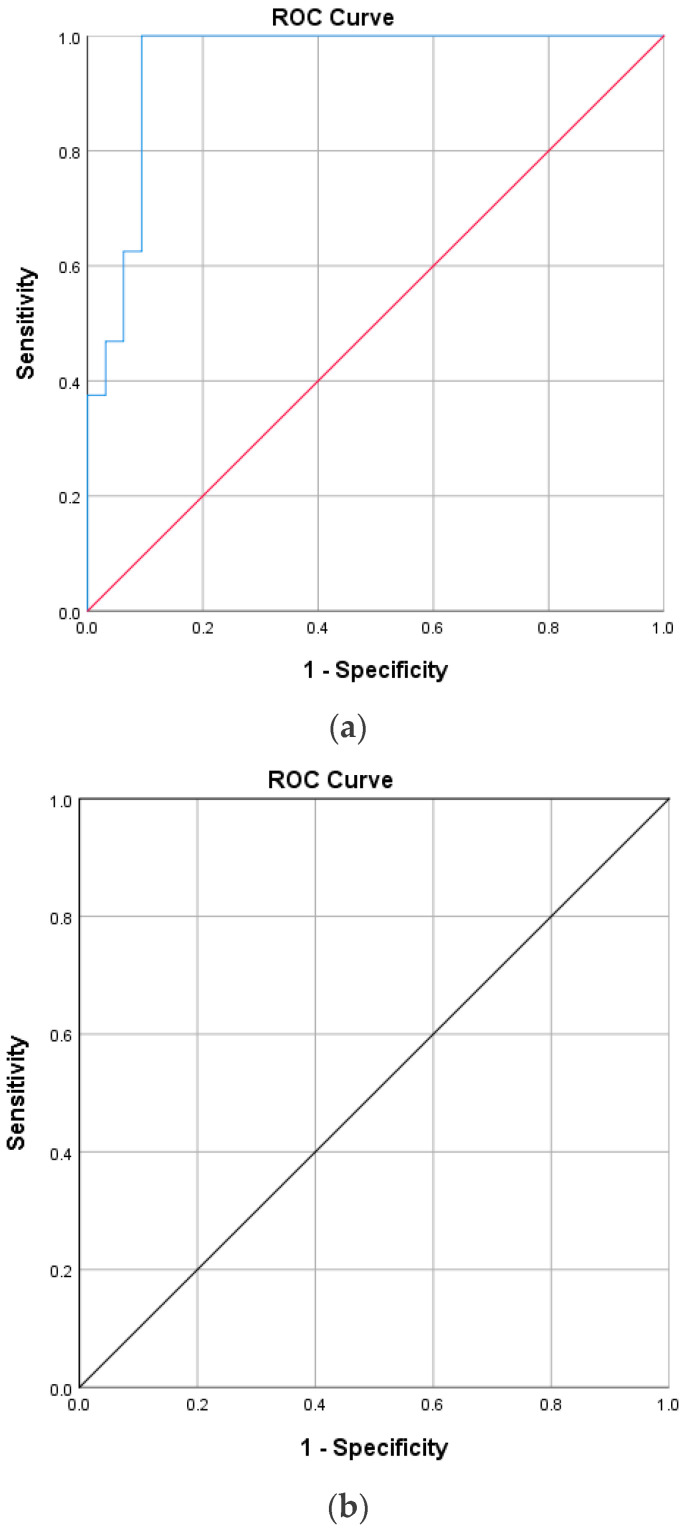
(**a**): Receiver operating characteristic curve for ellipsoid zone defect in diabetic macular edema patients (Blue line is the ROC curve and Red is the reference line). (**b**): Receiver operating characteristic curve for ellipsoid zone defect in macular hole patients.

The receiver operating characteristic (ROC) curves for ellipsoid zone defect in diabetic macular edema and macular hole patients are shown in Figure 3a,b.

ROC curve analysis showed that a residual EZ defect is an excellent structural biomarker for final BCVA, with an AUC of 0.95 for DME and 1.00 for FTMHs (*p* < 0.001) (Table 3 and Table 4).

The cut-off values for the residual ellipsoid zone thickness, determined using the ROC curve, are 72.2 microns for DME and 58.5 microns for FTMHs post-intervention, indicating good visual acuity.

The OCT slices for the two patient groups have been included as Supplementary Material in the manuscript. Specifically, Figure S1a and S1b depict diabetic macular edema group before and after intervention, respectively, while Figure S2a and S2b illustrate full-thickness macular hole group before and after intervention, respectively.

## 4. Discussion

Anti-VEGF therapy and successful MHS resulted in the notable restoration of the EZ, leading to improved BCVA following interventions.

In DME, OCT biomarkers serve as a useful tool for predicting anatomical and functional outcomes in response to anti-VEGF therapy. Panozzo et al. [16] analyzed seven parameters: foveal thickness (measured as either CST or mean volume), the size of intraretinal cysts, the status of the EZ and/or ELM, DRIL, hyperreflective foci, subfoveal fluid, and the vitreoretinal relationship. Saxena et al. demonstrated that mean CST, cube average thickness (CAT), and cube volume (CV) serve as independent markers of DME severity and prognostic factors for visual acuity [17]. Including multiple inter-related biomarkers, however, would have made it challenging to interpret and evaluate the influence of individual variables. As a result, we focused specifically on EZ changes and their direct correlation with visual acuity. Additionally, Santos et al. compared OCT baseline predictors and concluded that an intact EZ was the most reliable OCT marker of them all. Ellipsoid zone disruption and the disorganization of retinal inner layers are good predictors of treatment response after anti-VEGF therapy [18].

Campos et al. concluded that sensory neural detachment indicates an anatomic response, while an intact EZ signifies a functional response. However, the final BCVA depends not only on the resolution of retinal edema but also on the integrity of the photoreceptors [19]. The clinical determination of photoreceptor integrity relies on the assessment of the EZ, characterized by its elevated mitochondrial concentration that facilitates heightened energy utilization [20].

In our prior study, we observed a consecutive disruption of the ELM and the EZ. This underscored the essential prerequisite of an intact ELM for the preservation of an intact EZ [6,21]. Our recent research has shed light on the mechanism behind the restoration of the EZ following anti-VEGF therapy. It was observed that anti-VEGF therapy restores the barrier effect of ELM. It causes the ELM to be restored first, followed by EZ restoration [22]. Omri et al. [23] demonstrated that the ELM in rat and monkey retinas consists of connections between glial Muller cells and inner photoreceptor segments, with tight junctions (TJs) present. These TJs, having the protein occludin as a key protein, link Muller cells and photoreceptors at the ELM. Their study suggests that the ELM acts as part of the retinal barrier, which can be disrupted in certain diseases. In DR, glial Muller cells become swollen and lose occludin at the ELM, making these junctions potential targets for treatment. Murukami et al. [24] showed that VEGF treatment in primary bovine retinal endothelial cells causes occludin phosphorylation, ubiquitination, and the fragmentation of TJs, highlighting the key role of occludin in regulating barrier properties and its potential as a therapeutic target.

An earlier study found a direct correlation between the percentage of disruption in the EZ and VA in DME [25]. The preservation of EZ integrity has been identified as a favorable predictor for visual outcomes [26]. Chatzerelli et al. [27] similarly documented a substantial restoration of photoreceptors in the EZ among DME patients after 12 months of follow-up following intravitreal ranibizumab. The reduction in EZ defect size was influenced by the DME pattern observed in SD-OCT. Hareedy et al. [28] also found a notable correlation between the enhancement in BCVA and the status of the EZ following the second and third injections.

After successful macular hole surgery, ELM and EZ restoration begins, with ELM reconstruction occurring before the restoration of the EZ and foveal photoreceptor layer. Visual outcomes after MH closure are significantly better in eyes with a restored EZ compared to those with a disrupted EZ [29,30].

Our present study revealed a significant decline in logMAR VA in patients with noticeable EZ restoration. Specifically, EZ disruption was reduced from 73.4 to 19.4% after IVT and from 67 to 18.3% after MHS. The degree of EZ regeneration is believed to strongly influence the ultimate visual outcome. In the present study, there was a notable correlation between the final BCVA and the postoperative EZ defect. The AUC analysis underscored EZ regeneration as a highly effective structural biomarker for predicting visual recovery for both pharmacological and surgical interventions.

The effect of the restoration of the EZ and its effect on improvement in visual acuity is well known after anti-VEGF therapy and successful MHS. This study delves into the evaluation of the EZ as a structural biomarker after pharmacological and surgical interventions. The limitations of our study include its retrospective design, short follow-up period, and a small sample size without stratification by DME subtypes. However, this study is the first to highlight the EZ as a structural biomarker after pharmacological and surgical interventions for DME and FTMHs.

To conclude, the EZ proves to be a crucial structural biomarker for predicting visual outcomes in both pharmacological and surgical interventions.

## Figures and Tables

**Table 1:** Patient variables in diabetic macular edema.

Variables	Grade 0 (*n* = 4)	Grade 1 (*n* = 5)	Grade 2 (*n* = 23)	*p* Values
Age (years ± SD)	52.64 ± 2.85	53.20 ± 3.55	55.50 ± 4.29	<0.065
FBS (mg/dL)	89.66 ± 2.49	80.33 ± 4.18	89.61 ± 4.85	<0.001
PPBS (mg/dL)	132.50 ± 7.89	134.67 ± 5.18	127.50 ± 6.48	<0.001
HbA1c (%)	7.15 ± 1.53	6.98 ± 0.22	6.89 ± 0.37	<0.001
S. creatinine (mg/dL)	1.59 ± 0.21	1.51 ± 0.18	1.43 ± 0.15	<0.001
Pre-treatment CST range (µm)	385.93 ± 13.43	354.23 ± 15.00	346.89 ± 10.36	<0.001
Pre-treatment EZ defect (µm ± SD)	2014.40 ± 291.15	779.50 ± 121.50	434.00 ± 108.00	<0.001
Pre-treatment BCVA (logMar)	1.82 ± 0.37	1.54 ± 0.30	0.32 ± 0.17	<0.001
Post-treatment CST range (µm)	282.86 ± 8.85	278.52 ± 8.72	243.18 ± 7.88	<0.001
Post-treatment residual EZ defect (µm ± SD)	1778.67 ± 129.12	377.34 ± 71.32	00.00 ± 00.00	<0.001
Post-treatment BCVA (log Mar)	1.40 ± 0.60	0.75 ± 0.55	0.28 ± 0.09	<0.001

BCVA: best-corrected visual acuity, FBS: fasting blood sugar, PPBS: post-prandial blood sugar, HbA1c: glycosylated hemoglobin, EZ: ellipsoid zone, CST: central subfoveal thickness, SD: standard deviation.

**Table 2:** Patient variables in full-thickness macular holes.

Variables	Grade 0 (*n* = 4)	Grade 1 (*n* = 7)	Grade 2 (*n* = 21)	*p* Values
Age (years ± SD)	52.50 ± 5.99	56.20 ± 3.29	53.64 ± 2.99	<0.053
Minimum linear diameter (µm ± SD)	544.53 ± 75.90	512.26 ± 52.32	444.53 ± 65.85	<0.001
Base diameter (µm ± SD)	931.54 ± 121.00	810.90 ± 115.26	693.35 ± 98.14	<0.001
Pre-treatment EZ defect (µm ± SD)	2231.36 ± 172.11	1573.93 ± 189.55	759.52 ± 119.05	<0.001
Pre-treatment BCVA (logMar)	1.40 ± 0.39	1.17 ± 0.16	1.14 ± 0.19	<0.001
Post-treatment residual EZ defect (µm ± SD)	1669.49 ± 51.76	111.70 ± 52.26	0.00	<0.001
Post-treatment BCVA (logMar)	1.12 ± 0.16	0.49 ± 0.08	0.24 ± 0.08	<0.001

BCVA: best-corrected visual acuity, EZ: ellipsoid zone, SD: standard deviation. Minimum linear diameter: the narrowest distance between the rims of the MH, parallel to the retinal pigment epithelium. Base diameter: the maximum diameter of the hole’s base area, which is the area of the hole in the plane of the retinal pigment epithelium.

**Table 3:** Area under the curve for ellipsoid zone defect in diabetic macular edema patients.

Area Under the Curve				
Test Result Variable: Ellipsoid Zone Defect				
Area	Std. Error ^a^	Asymptotic Sig. ^b^	Asymptotic 95% Confidence Interval	
			Lower Bound	Upper Bound
0.952	0.028	0.000	0.897	1.000

The ROC curve analysis for discrimination included cohorts divided into Grades 0 (*n* = 4), 1 (*n* = 5), and 2 (*n* = 23) on the basis of the EZ defect for DME. ^a^. Under the nonparametric assumption; ^b^. null hypothesis: true area = 0.5.

**Table 4:** Area under the curve for ellipsoid zone defect in macular hole patients.

Area Under the Curve				
Test Result Variable: Ellipsoid Zone Defect				
Area	Std. Error ^a^	Asymptotic Sig. ^b^	Asymptotic 95% Confidence Interval	
			Lower Bound	Upper Bound
1.000	0.017	0.000	1.000	1.000

The ROC curve analysis included cohorts divided into Grades 0 (*n* = 4), 1 (*n* = 7), and 2 (*n* = 21) on the basis of the EZ defect for MHS. ^a^. Under the nonparametric assumption; ^b^. null hypothesis: true area = 0.5.

## Data Availability

The original contributions presented in the study are included in this article and Supplementary Material, further inquiries can be directed to the corresponding authors.

## Supplementary Materials

The following supporting information can be downloaded at: https://www.mdpi.com/article/10.3390/vision9010004/s1, Figure S1a: SD-OCT image shows diabetic macular edema, pre-intervention, Figure S1b: SD-OCT image shows diabetic macular edema, 12th week post intervention with restored ellipsoid zone defect (arrow) and external limiting membrane (arrow head), Figure S2a: SD-OCT image shows full thickness macular hole, pre-intervention. The minimum linear diameter, basal diameter and ellipsoid zone defect have been marked (top to bottom), Figure S2b: SD-OCT image shows full thickness macular hole closure at 12th week post intervention with residual minimal ellipsoid zone defect (arrow).
