# Supplementary material for: The Ellipsoid Zone Is a Structural Biomarker for Visual Outcomes in Diabetic Macular Edema and Macular Hole Management
Source: Vision (Basel). 2025 Jan 13;9(1):4. doi: 10.3390/vision9010004 (PMC11755456; doi:10.3390/vision9010004)
Supplement: Supplementary file 1 [file vision-09-00004-s001.zip › vision-3207299-supplementary.pdf]

SUPPLEMENTARY MATERIAL

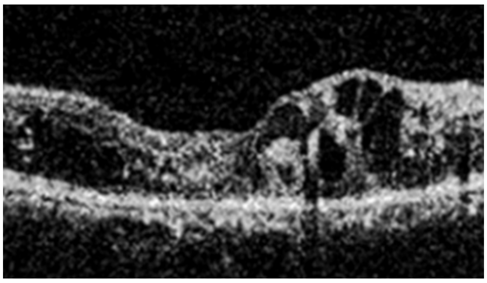

Figure S1 (a): SD-OCT image shows diabetic macular edema, pre-intervention.

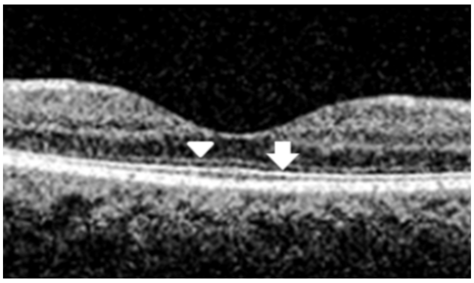

Figure S1 (b): SD-OCT image shows diabetic macular edema,12th week post intervention with restored ellipsoid zone defect (arrow) and external limiting membrane (arrow head).

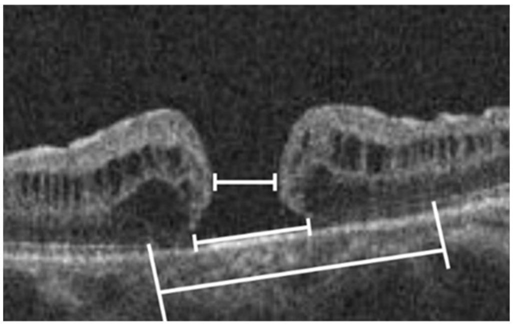

Figure S2 (a): SD-OCT image shows full thickness macular hole, pre-intervention. The minimum linear diameter, basal diameter and ellipsoid zone defect have been marked (top to bottom)

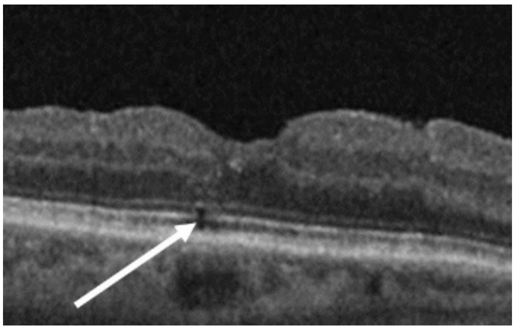

Figure S2 (b): SD-OCT image shows full thickness macular hole closure at 12th week post intervention with residual minimal ellipsoid zone defect (arrow).
